# Combined influence of basal media and fibroblast growth factor on the expansion and differentiation capabilities of adipose-derived stem cells

**DOI:** 10.1186/2045-9769-3-13

**Published:** 2014-11-26

**Authors:** Mark Ahearne, Joanne Lysaght, Amy P Lynch

**Affiliations:** 1 Trinity Centre for Bioengineering, Trinity Biomedical Sciences Institute, Trinity College Dublin, Dublin, Ireland; 2 Department of Mechanical and Manufacturing Engineering, School of Engineering, Trinity College Dublin, Dublin, Ireland; 3 Department of Surgery, Institute of Molecular Medicine, Trinity Centre for Health Sciences, St. James’s Hospital, Dublin 8, Ireland

**Keywords:** Cell differentiation, Cartilage, Bone, Adipose, Cornea

## Abstract

**Background:**

Interest in adipose-derived stem cells (ASCs) has increased in recent years due to their multi-linage differentiation capabilities. While much work has been done to optimize the differentiation media, few studies have focused on examining the influence of different expansion media on cell behavior. In this study, three basal media (low glucose Dulbecco’s modified Eagle’s medium (DMEM), high glucose DMEM and DMEM-F12) supplemented with or without fibroblast growth factor 2 (FGF) were examined to assess their suitability for expanding ASCs.

**Findings:**

Flow cytometry, colony-forming unit assays (CFU-Fs) and differentiation assays were utilized to study cell behavior. High glucose media CFU-Fs produced fewest colonies while the addition of FGF increased colony size. By passage 2, the majority of cells were positive for CD44, 45, 73, 90 and 105 and negative for CD14, 31 and 45, indicating a mesenchymal phenotype. A sub-population of CD34 positive cells was present among passage 2 cells; however, by passage 4 the cells were negative for CD34. FGF has a negative effective on passage 4 ASC adipogenesis and high glucose media plus FGF-enhanced osteogenic capacity of passage 4 ASCs. FGF supplemented basal media were most suitable for chondrogenesis. High glucose media plus FGF appeared to be the most beneficial for priming ASCs to induce a keratocyte phenotype.

**Conclusions:**

These findings demonstrate the reciprocal effect FGF and basal media have on ASCs. This research has implications for those interested regenerating bone, cartilage, cornea or adipose tissues.

## Findings

### Research hypothesis

The ability of adipose-derived stem cells (ASCs) to undergo prolonged periods of expansion and differentiation towards specific lineages has made them a valuable cell source for clinicians and researchers interested in tissue repair and regenerative medicine. These cells are considered to exhibit mesenchymal stem cell characteristics by their ability to form bone, cartilage and adipose tissues and by the presence or absence of specific CD markers [1]. Maintaining a suitable culture environment is essential for retaining the stem-like characteristics of ASCs. Commercially available media have been formulated to cultivate these cells; however, these are generally more expensive than standard culture media and their effectiveness over standard chemically defined supplemented media has been brought into question [2]. For these reasons, many researchers continue to use media consisting of Dulbecco’s modified Eagle’s medium (DMEM) supplemented with fetal bovine serum (FBS), antibiotics and in some cases growth factors most commonly fibroblast growth factor 2 (FGF). Low glucose DMEM [3, 4], high glucose DMEM [5, 6] and DMEM plus nutrient mix F12 [7, 8] are among the most commonly used basal media for expanding ASCs, although many publications do not state the media glucose concentration. There appears to be little consensus as to which of these media sources and growth factors are most suitable for ASC expansion. The aims of this study were to compare three of the most commonly used basal media for ASC expansion (low glucose DMEM, high glucose DMEM and DMEM-F12) and determine whether they had any advantages or limitations. The influence of adding FGF to each of these media was also examined. After expansion in each type of media, the cells were placed in chemically defined differentiation media to promote an adipogenic, osteogenic, chondrogenic or keratocyte phenotype. ASC differentiation was evaluated and used to determine the most effective expansion media for each specific lineage.

## Materials and methods

### Isolation and culture of adipose-derived stem cells

Subcutaneous adipose tissue was taken with consent from patients undergoing esophageal surgery. This study has received ethical approval from the St. James’s Hospital Institutional Review Board. The tissue was then digested by using 300 unit/ml collagenase type I solution under constant agitation for 90 min at 37°C. Once digested, the tissue was poured through a 100 μm filter and centrifuged to separate the stromal vascular fraction from the adipocytes. The adipose stromal vascular fraction was then suspended in erythrocyte lysis buffer consisting of 155 mM NH_4_Cl, 10 mM K_2_CO_3_ and 0.1 mM EDTA in H_2_O at room temperature for 10 min. The cells were centrifuged, suspended in fresh media and poured through a 40 μm filter. The cells were then counted and split into six groups: 1) low glucose DMEM (LG) with a glucose concentration of 1,000 mg/l; 2) high glucose DMEM (HG) with a glucose concentration of 4,500 mg/l; 3) DMEM-F12 (F12); 4) low glucose DMEM supplemented with FGF (LG + FGF); 5) high glucose DMEM supplemented with FGF (HG + FGF) and 6) DMEM-F12 supplemented with FGF (F12 + FGF). All basal media were purchased from Thermo Scientific (Hyclone). All media were supplemented with 10% (*v*/*v*) FBS (Hyclone, Thermo Scientific), 100 U/ml penicillin (Gibco), 100 μg/ml streptomycin (Gibco) and 250 ng/ml amphotericin-B (Sigma-Aldrich). When FGF was added, it gave a final concentration of 10 ng/ml in media. Cells isolated from two separate donors were examined.

### Colony-forming unit assay (CFU-F)

Freshly isolated cells from the stromal fraction were plated onto Petri dishes at a concentration of 50 cells/cm^2^. These cells were cultured in one of the six media already mentioned (LG, HG, F12, LG + FGF, HG + FGF and F12 + FGF) for 14 days. The cells were then washed using PBS, fixed in 2% (*w*/*v*) paraformaldehyde and stained using 1% (*w*/*v*) crystal violet solution. The numbers of colonies formed on each dish were counted.

### Flow cytometry

Passage 2 (P2) and passage 4 (P4) ASCs that had been cultured in all six media types were suspended in a buffer solution consisting of PBS with 2% FBS. The cells were labelled using fluorescently conjugated primary antibodies for CD14, 19, 31, 34, 44, 45, 73, 90, 105 and 146 (all eBioscience) in buffer solution as previously described [9]. The cells were then repeatedly washed and centrifuged to remove unbound antibodies. Compensation controls were performed using compensation beads (eBioscience). Flow cytometry was performed using a LSRFortessa flow cytometer (BD Biosciences). The data collected was analysed using Flowing Software version 2.5.1 (University of Turku, Finland).

### Differentiation assays

For adipogenesis, 5 × 10^4^ P2 or P4 ASCs cultured in each of the six media types were plated onto each well of a six-well plate and cultured in their respective expansion media at 37°C, 5% CO_2_ until they reached 80% confluence. The cells were then cultured for a further 21 days in an adipogenic differentiation media. After 21 days in culture the cells were fixed using 4% (*w*/*v*) paraformaldehyde and then stained using 1% (*w*/*v*) oil red O solution. Images were recorded using a light microscope at six random locations in each well. The percentage surface area stained positive by the oil red O in each well was quantified using ImageJ software (NIH). Cells cultured in regular expansion media were used as a negative control. For osteogenesis, 5 × 10^4^ P2 or P4 ASCs were plated onto each well of a six-well plate and cultured in their respective expansion media at 37°C, 5% CO_2_ until they reach 80% confluence. The cells were then cultured for a further 21 days in an osteogenic differentiation media [9]. After 21 days, the cells were fixed using ice-cold ethanol and stained using 1% (*w*/*v*) alizarin red solution for 2 min. The plates were then washed until the negative control wells were clear. Images of the staining were recorded. The absorbance of each well was measured at a wavelength of 405 nm to determine the intensity of alizarin red staining. For chondrogenesis, P2 or P4 ASCs were centrifuged to form pellets each containing 250,000 cells. The pellets were cultured in a chondrogenic differentiation media [10]. The pellets were cultured at 37°C, 5% CO_2_, 5% O_2_ for 21 days. The pellets were then taken for biochemical analysis or fixed in 4% (*w*/*v*) paraformaldehyde. Fixed pellets were dehydrated using alcohol and zylene, embedded in paraffin wax. The pellets were then sliced (5 μm) and stained for GAG and cells using alcian blue and 0.1% (*w*/*v*) nuclear fast red solution. Pellets for biochemical analysis were digested using a papain digest for 18 h at 60°C. DNA was quantified using a Hoechst 33324 assay while sGAG was quantified using a DMMB assay as previously described [5].

For keratocyte differentiation, 250,000 P2 cells were seeded to 25-cm^2^ culture flasks and cultured for 14 days in a previously defined keratocyte differentiation media [11] consisting of advanced DMEM (Gibco) supplemented with 10 ng/ml FGF, 0.1 mM/ml L-ascorbic acid-2-phosphate (Sigma-Aldrich) and 2 mM/ml GlutaMax (Gibco) with the addition of 100 U/ml penicillin, 100 μg/ml streptomycin and 250 ng/ml amphotericin-B. Real-time reverse transcription polymerase chain reaction (RT-PCR) was used to quantify relative gene expression. Cellular RNA was extracted from cells using 1 ml of TRIZOL (Invitrogen). Chloroform (200 μl) was added and the solution centrifuged. The supernatant was removed and isopropanol added. Following overnight incubation at -20°C, the solution was centrifuged and supernatant discarded. Ethanol (70%) was added, the solution centrifuged and the ethanol removed. The remaining pellet was suspended in RNAse free water. A high capacity cDNA reverse transcription kit (Invitrogen) was used to reverse transcribe RNA using a thermocycler. Real-time PCR of the resultant cDNA was performed using TaqMan gene expression assay primers and TaqMan Universal Master Mix II (all Applied Biosystems). The primers examined were glyceraldehyde-3-phosphate dehydrogenase (GAPDH, Hs02758991_g1), aldehyde dehydrogenases 3A1 (ALDH3A1, Hs00964880_m1), smooth muscle actin (αSMA/ACTA2, Hs00426835_g1), collagen type I (COL1A1, Hs00164004_m1) and collagen type III (COL3A1, Hs00943809_m1). Each gene of interest was normalized against GAPDH using the ΔΔCt method. Calculated values were expressed as a power of 2^-ΔΔCt^. For this study, all values were then normalized to the LG group of samples.

### Statistical analysis

Data was analysed using GraphPad Prism 5. Two-way ANOVA accompanied by a Tukey multiple comparison test or Bonferroni post-test was used to analyse data. Significance was accepted at a level of *p* < 0.05.

## Results and discussion

**Figure 1:**
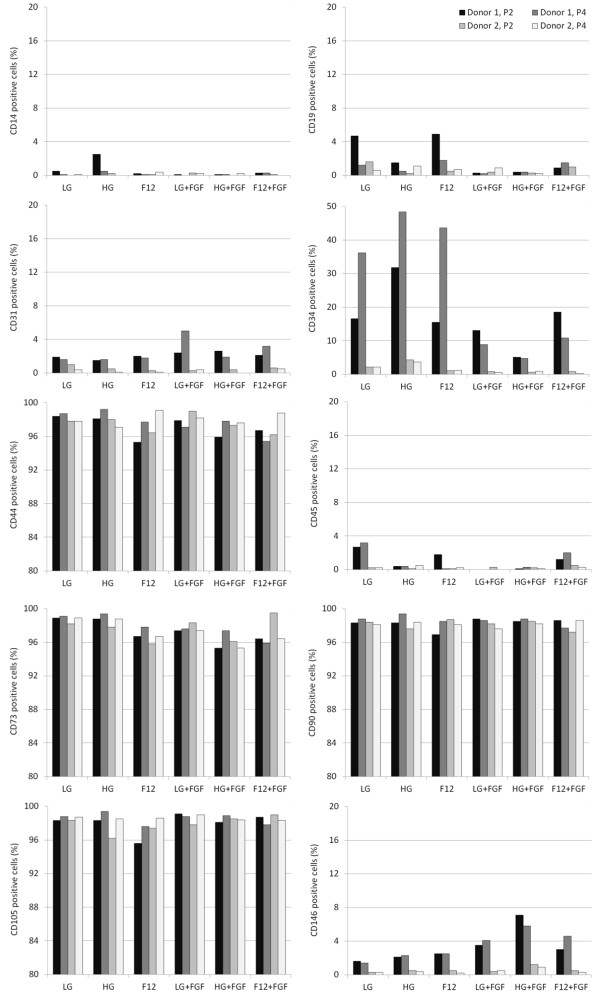
**Percentage of P2 and P4 ASCs isolated from two different donors that were positive for CD14**, **CD19**, **CD31**, **CD34**, **CD44**, **CD45**, **CD73**, **CD90**, **CD105** **and CD146.**

All the ASCs were positive and negative for appropriate markers normally associated with mesenchymal stem cells apart from CD34 where there was a separate population of cells at P2 expressing this marker, the size of which varied depending on the donor and the type of culture media used (Figure 1). By passage 4, the majority of cells were CD34 negative. CD34 is most commonly used as a hematopoietic progenitor and endothelial cell marker [12, 13]; although given the almost complete lack of CD45 and CD31 positive cells, these effectively eliminate the presence of CD34 positive hematopoietic and endothelial cells among the cell population. While it has generally been believed that expanded mesenchymal stem cells are negative for CD34 [1], its presence has been found among freshly isolated mesenchymal stem cell populations [14]. Among adipose derived cells, the percentage of CD34 positive cells has been shown to decrease with culture time and may indicate an immature phenotype prior to differentiation towards a specific linage [15] although from our findings there was no obvious correlation between the size of the population of CD34 positive cells and their differential capabilities. In this study, the cells at P2 had undergone 4 to 5 population doublings while those cells at P4 had undergone 10 to 12 population doublings although this assumes that all cells initially seeded are ASCs and all cells proliferate at the same rate.

**Figure 2:**
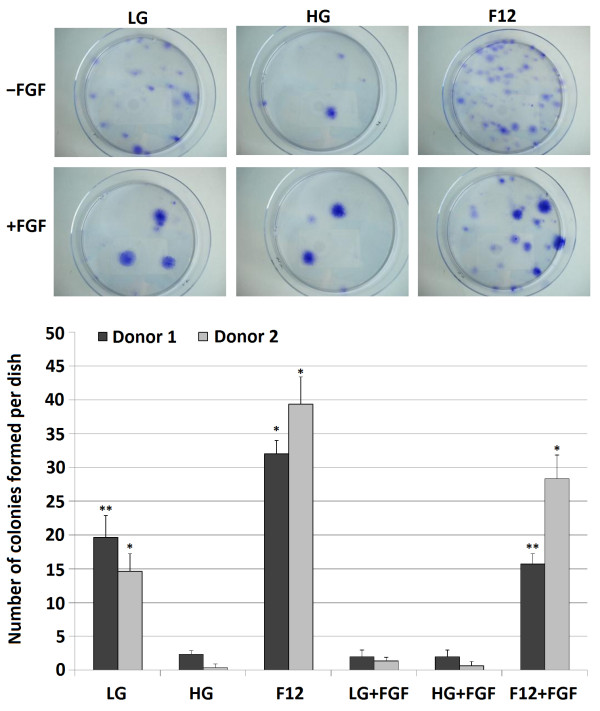
**CFU-F assays showing the number of colonies formed after 14 days from freshly isolated cells from stromal vascular fraction of adipose tissue cultured under different media conditions.** *n* = 3 dishes counted per samples, *significant difference with all other groups, **significant different with HG, F12, LG + FGF and HG + FGF, *p* < 0.05.

The number and size of colonies formed from freshly isolated ASCs in CFU-Fs varied considerably when using different media types (Figure 2). F12 and LG produced significantly more colonies than HG media. Supplementation with FGF resulted in fewer colonies in F12 and LG media, but much larger colonies were formed. Normally, increasing the number of colonies would suggest a higher proportion of stem cells among the freshly isolated cell population. However, since the same initial number of cells was used in all groups, this demonstrates that the culture conditions also affect colony formation and expansion.

**Figure 3:**
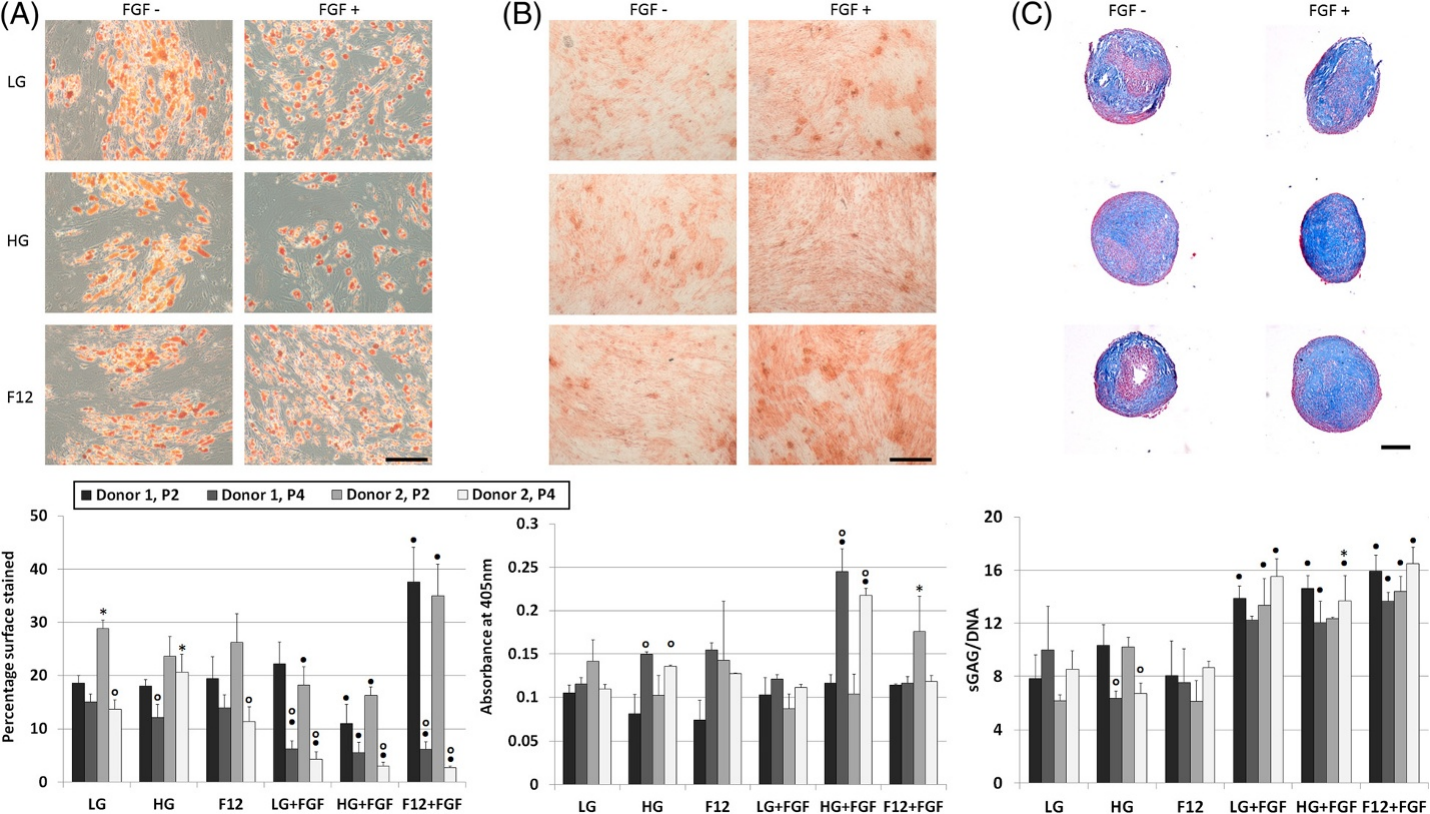
**P2 and P4 ASCs cultured from two different donors in chemically defined differentiation media after 21 days. (A)** Adipogenic assay stained using oil red O and quantified by measuring the percentage of surface area stained; **(B)** osteogenic assay stained using alizarin red and quantified by measuring the light absorbance at 405 nm; **(C)** chondrogenic pellet assay stained using alcian blue and nuclear fast red and quantified using biochemical assays for DNA and GAG. *n* = 3, **●** represents a significant difference between FGF and the equivalent non-FGF-supplemented media; o represents a significant difference between P2 and P4 cells; *significant difference between donors, *p* < 0.05; scalebar = 20 μm for A and B and 100 μm for C, representative images display the results from P2 donor 1.

The findings of this study would suggest that choosing the optimal media for expanding ASCs is dependent on the final application. The addition of FGF was found to cause a significant increase in adipogenesis (Figure 3A) at P2 when added to F12 media (*p* < 0.05). By P4, cells expanded in FGF-supplemented media showed a decrease in adipogenesis regardless of the basal media. The expansion media had no significant effect on the osteogenic potential of P2 cells although HG + FGF-cultured cells were significantly better at undergoing osteogenesis than cells expanded in the other media at P4 (Figure 3B). Cells expanded in FGF-supplemented media displayed a higher chondrogenic capacity when compared to cells expanded in media without FGF regardless of the basal media used (Figure 3C). The center of pellets formed from cells that has not been pre-cultured in FGF appeared to contain substantially less sGAG than FGF-expanded cells. These findings would correlate with several other studies which have found that FGF primes cells, thus enabling them to become more chondrogenic when moved to a chemically defined differentiation media [16, 17].

**Figure 4:**
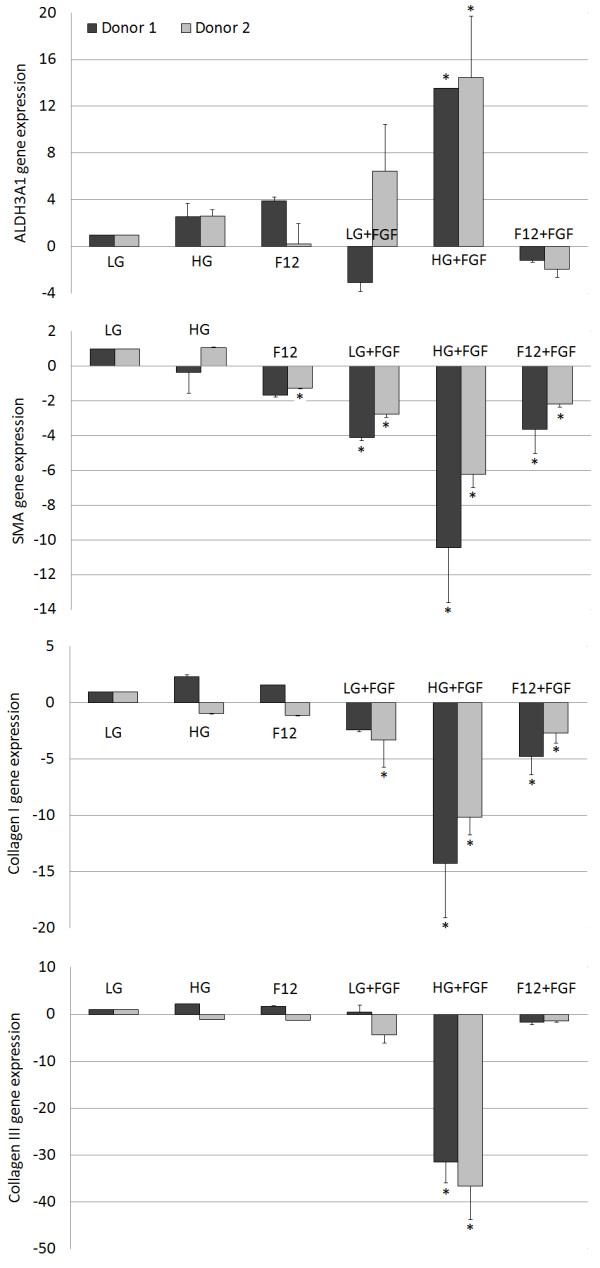
**ALDH3A1**, **αSMA**, **collagen I and collagen III gene expressions of P2 ASCs cultured from two different donors relative to LG after culture for 14 days in keratocyte differentiation media.** *n* = 3, *significant difference relative to LG, *p* < 0.05.

Following culture in a keratocyte differentiation media for 14 days, ASCs cultured in HG + FGF expressed the highest levels of the keratocyte marker ALDH3A1 lowest levels of the fibrotic markers alpha smooth muscle actin (αSMA/ACTA2), collagen I (COL1A1) and collagen III (COL3A1) (Figure 4). Keratocytes are quiescent cells native to the corneal stroma that are believed to be derived from the neural crest [18] and are characterized by the presence of ALDH3A1 and the absence of αSMA/ACTA2. Upon injury to the cornea, keratocytes become activated and may exhibit fibroblastic or myofibroblastic characteristics. These are normally associated with the loss of corneal transparency and the formation of fibrotic scar tissue. Fibroblastic or myofibroblastic activity result in a downregulation of ALDH3A1 and upregulation of COL1A1, COL3A1 and αSMA/ACTA2 [19, 20]. Gene expression analysis in this study suggests that ASCs expanded in HG + FGF had the most similar phenotype to native quiescent corneal keratocytes.

While several previous studies have examined the effect of FGF or different basal media on the expansion and differentiation of ASCs [17, 21, 22] or cells from other sources [23–25], this is the first study we are aware of that has examined the combined influence of FGF and different basal media on ASC behavior. While the specific molecular mechanism by which each media type affects ASC behavior is not fully understood, it is clear that the choice of media plays a key role in determining the final differential capabilities of the cells. Glucose for example is known to enhance nucleotide synthesis that in turn regulates stem cell fate [26]. FGF has been shown to upregulate transcription factor SOX2 and inhibit Wnt signaling which subsequently leads to a reduced osteogenic differentiation capacity [27], while inhibition of Wnt has also been shown to promote the early stages of chondrogenesis of mesenchymal stem cells [28]. Examining the influence that different biochemical factors have on cell behavior is vital in determining the most suitable media formulation for a particular application. Our findings demonstrate the effect that basal media and growth factors can have on the cells characteristics when expanding ASCs and their subsequent differentiation towards specific lineages.

## Abbreviations

ALDH3A1: Aldehyde dehydrogenases 3A1
αSMA: alpha smooth muscle actin
ASC: Adipose derived stem cell
CFU-F: Colony-forming unit assay
COL1A1: Collagen type I
COL3A1: Collagen type III
DMEM: Dulbecco’s modified Eagle’s medium
FBS: Fetal bovine serum
FGF: Fibroblast growth factor 2
F12: DMEM plus nutrient mix F12
GAPDH: Glyceraldehyde-3-phosphate dehydrogenase
HG: High glucose media
LG: Low glucose media
P2: Passage 2
P4: Passage 4
RT-PCR: Real-time reverse transcription polymerase chain reaction
SOX2: Sex determining region Y box 2.

## References

[1] Dominici M, Le Blanc K, Mueller I, Slaper-Cortenbach I, Marini F, Krause D, Deans R, Keating A, Prockop D, Horwitz E (2006). Minimal criteria for defining multipotent mesenchymal stromal cells. The International Society for Cellular Therapy position statement. Cytotherapy.

[2] Baer PC, Griesche N, Luttmann W, Schubert R, Luttmann A, Geiger H (2010). Human adipose-derived mesenchymal stem cells in vitro: evaluation of an optimal expansion medium preserving stemness. Cytotherapy.

[3] Francis MP, Sachs PC, Elmore LW, Holt SE (2010). Isolating adipose-derived mesenchymal stem cells from lipoaspirate blood and saline fraction. Organogenesis.

[4] Faroni A, Rothwell SW, Grolla AA, Terenghi G, Magnaghi V, Verkhratsky A (2013). Differentiation of adipose-derived stem cells into Schwann cell phenotype induces expression of P2X receptors that control cell death. Cell Death Dis.

[5] Ahearne M, Kelly DJ (2013). A comparison of fibrin, agarose and gellan gum hydrogels as carriers of stem cells and growth factor delivery microspheres for cartilage regeneration. Biomed Mater.

[6] Dhanasekaran M, Indumathi S, Rajkumar JS, Sudarsanam D (2013). Effect of high glucose on extensive culturing of mesenchymal stem cells derived from subcutaneous fat, omentum fat and bone marrow. Cell Biochem Funct.

[7] Estes BT, Diekman BO, Gimble JM, Guilak F (2010). Isolation of adipose-derived stem cells and their induction to a chondrogenic phenotype. Nat Protoc.

[8] Balwierz A, Czech U, Polus A, Filipkowski RK, Mioduszewska B, Proszynski T, Kolodziejczyk P, Skrzeczynska-Moncznik J, Dudek W, Kaczmarek L, Kulig J, Pryjma J, Dembinska-Kiec A (2008). Human adipose tissue stromal vascular fraction cells differentiate depending on distinct types of media. Cell Prolif.

[9] Ahearne M, Liu Y, Kelly DJ (2014). Combining freshly isolated chondroprogenitor cells from the infrapatellar fat pad with a growth factor delivery hydrogel as a putative single stage therapy for articular cartilage repair. Tissue Eng Part A.

[10] Ahearne M, Buckley CT, Kelly DJ (2011). A growth factor delivery system for chondrogenic induction of infrapatellar fat pad-derived stem cells in fibrin hydrogels. Biotechnol Appl Biochem.

[11] Du Y, Roh DS, Funderburgh ML, Mann MM, Marra KG, Rubin JP, Li X, Funderburgh JL (2010). Adipose-derived stem cells differentiate to keratocytes in vitro. Mol Vis.

[12] Fina L, Molgaard HV, Robertson D, Bradley NJ, Monaghan P, Delia D, Sutherland DR, Baker MA, Greaves MF (1990). Expression of the CD34 gene in vascular endothelial cells. Blood.

[13] Young PE, Baumhueter S, Lasky LA (1995). The sialomucin CD34 is expressed on hematopoietic cells and blood vessels during murine development. Blood.

[14] Lin CS, Ning H, Lin G, Lue TF (2012). Is CD34 truly a negative marker for mesenchymal stromal cells?. Cytotherapy.

[15] Suga H, Matsumoto D, Eto H, Inoue K, Aoi N, Kato H, Araki J, Yoshimura K (2009). Functional implications of CD34 expression in human adipose-derived stem/progenitor cells. Stem Cells Dev.

[16] Handorf AM, Li WJ (2011). Fibroblast growth factor-2 primes human mesenchymal stem cells for enhanced chondrogenesis. PLoS One.

[17] Buckley CT, Kelly DJ (2012). Expansion in the presence of FGF-2 enhances the functional development of cartilaginous tissues engineered using infrapatellar fat pad derived MSCs. J Mech Behav Biomed Mater.

[18] Bard JB, Hay ED (1975). The behavior of fibroblasts from the developing avian cornea. Morphology and movement in situ and in vitro. J Cell Biol.

[19] Funderburgh JL, Mann MM, Funderburgh ML (2003). Keratocyte phenotype mediates proteoglycan structure: a role for fibroblasts in corneal fibrosis. J Biol Chem.

[20] Pei Y, Reins RY, McDermott AM (2006). Aldehyde dehydrogenase (ALDH) 3A1 expression by the human keratocyte and its repair phenotypes. Exp Eye Res.

[21] Dhanasekaran M, Indumathi S, Rashmi M, Rajkumar JS, Sudarsanam D (2012). Unravelling the retention of proliferation and differentiation potency in extensive culture of human subcutaneous fat-derived mesenchymal stem cells in different media. Cell Prolif.

[22] Kabiri A, Esfandiari E, Hashemibeni B, Kazemi M, Mardani M, Esmaeili A (2012). Effects of FGF-2 on human adipose tissue derived adult stem cells morphology and chondrogenesis enhancement in Transwell culture. Biochem Biophys Res Commun.

[23] Aguiari P, Leo S, Zavan B, Vindigni V, Rimessi A, Bianchi K, Franzin C, Cortivo R, Rossato M, Vettor R, Abatangelo G, Pozzan T, Pinton P, Rizzuto R (2008). High glucose induces adipogenic differentiation of muscle-derived stem cells. Proc Natl Acad Sci U S A.

[24] Hagmann S, Moradi B, Frank S, Dreher T, Kammerer PW, Richter W, Gotterbarm T (2013). FGF-2 addition during expansion of human bone marrow-derived stromal cells alters MSC surface marker distribution and chondrogenic differentiation potential. Cell Prolif.

[25] Ramasamy R, Tong CK, Yip WK, Vellasamy S, Tan BC, Seow HF (2012). Basic fibroblast growth factor modulates cell cycle of human umbilical cord-derived mesenchymal stem cells. Cell Prolif.

[26] Oburoglu L, Tardito S, Fritz V, de Barros SC, Merida P, Craveiro M, Mamede J, Cretenet G, Mongellaz C, An X, Klysz D, Touhami J, Boyer-Clavel M, Battini JL, Dardalhon V, Zimmermann VS, Mohandas N, Gottlieb E, Sitbon M, Kinet S, Taylor N (2014). Glucose and glutamine metabolism regulate human hematopoietic stem cell lineage specification. Cell Stem Cell.

[27] Mansukhani A, Ambrosetti D, Holmes G, Cornivelli L, Basilico C (2005). Sox2 induction by FGF and FGFR2 activating mutations inhibits Wnt signaling and osteoblast differentiation. J Cell Biol.

[28] Im GI, Quan Z (2010). The effects of Wnt inhibitors on the chondrogenesis of human mesenchymal stem cells. Tissue Eng Part A.

